# Celiac Artery Compression Syndrome in an Adolescent Male

**DOI:** 10.7759/cureus.60580

**Published:** 2024-05-19

**Authors:** Data Don-Pedro

**Affiliations:** 1 Pediatrics, Omni Family Health, California, USA

**Keywords:** adolescent medicine, pediatric vascular/cardiology, the median arcuate ligament compresses the celiac trunk, dunbar syndrome, celiac artery compression syndrome

## Abstract

Celiac artery compression syndrome is not frequent in the pediatric population. The syndrome may entail long-standing abdominal pain, recurrent vomiting, bloating, weight loss, and an abdominal bruit, which in the case of our patient, was an incidental finding. Notably, patients may be asymptomatic.

Our patient is a 16-year-old male who presented with concerns about multiple, non-tender chest lymph nodes lasting for two weeks. He had also lost 80 lbs. over one year. On examination, however, an abdominal bruit was discovered, and a diagnostic workup was significant for celiac artery compression following a magnetic resonance angiography of the abdomen. Due to his significant weight loss and mediastinal lymphadenopathy, a chest computed tomography (CT) scan was done to rule out malignancy. The chest CT scan was reported as normal. Additionally, a renal duplex ultrasound was done to rule out renal artery stenosis, considering he had presented with elevated blood pressure; this was also unremarkable.

Although this patient had a history of marijuana use, his assessment did not show marked dependence. Substance abuse and atherosclerotic vascular disease can be predisposing factors for celiac artery compression syndrome in older individuals. However, compression of the celiac trunk by the median arcuate ligament is a congenital anomaly more appreciated in younger age groups. The patient was referred to vascular surgery for possible median arcuate ligament release.

## Introduction

Celiac artery compression syndrome (CACS) is a rare condition caused by the compression of the celiac artery by the median arcuate ligament, which is a fibrous band of the diaphragm [1]. It is also known as median arcuate ligament syndrome (MALS)[1]. The median arcuate ligament is a fibrous arch that traverses the aorta and bridges the crura of the diaphragm [1]. CACS can occasionally cause postprandial abdominal pain, with an incidence of 2 cases/100,000 [2].

The celiac trunk usually stems out from the abdominal aorta below the median arcuate ligament (between T11 and L1). However, its location of origin may vary [1,3]. A superior or inferior origin of the celiac axis can make it prone to compression [1,3]. Approximately 10% of the affected individuals have an abnormally positioned median arcuate ligament that appears to compress an otherwise normally positioned celiac artery [1,4]. CACS is more prevalent in females than males (4:1 ratio) between the ages of 40-60 years with a thin body habitus [1,5,6]. Hence, this case, occurring in the pediatric population, is an interesting outlier.

## Case presentation

Our patient is a 16-year-old male with multiple, tiny, painless chest masses for two weeks. There was no history of chronic cough, recent travels, night sweats, or fevers. However, he did report an intentional weight loss of ~80 lbs. over the past year due to “dietary and lifestyle” modifications. He reported a one-day history of vague abdominal pain and bloating a day before presentation but denied recurrence. The abdominal pain was not aggravated by position or diet. There was no nausea, vomiting, or diarrhea. He had Grade 3 acne, and other reviews of pertinent systems were unremarkable. There was a positive family history of idiopathic pulmonary fibrosis in a first-degree relative, but no known history of malignancies. The patient reported past marijuana use (with a CRAFFT score of 2/6), which he said he was actively quitting. CRAFFT is an acronym used as a tool for substance abuse screening in adolescents; for which 1 point is given to a "Yes" response and 0 to a "No" response (Table 1) [7]. 

**Table 1:** CRAFFT scoring for Substance abuse screening CRAFFT scoring is based on the American Academy of Pediatrics Bright Futures guidelines for primary care settings.

Acronym	Question	Patient’s response
C	Have you ever ridden in a car driven by someone under the influence of drugs or alcohol?	No
R	Do you ever use alcohol or drugs to relax, and feel better?	Yes
A	Do you ever use alcohol or drugs alone?	Yes
F	Do you ever forget things you did while using alcohol or drugs?	No
F	Do your family or friends ever tell you that you should cut down on your drinking or drug abuse?	No
T	Have you ever gotten into trouble while you were using alcohol or drugs?	No

On examination, the patient appeared well nourished with a body mass index (BMI) in the 84th percentile. His brachial systolic blood pressure (BP) was in the 99th percentile and his diastolic BP was in the 97th percentile for his height, age, and sex. His femoral-brachial systolic BP difference was significant at 40 mmHg. Multiple non-tender, firm, inter-mammary lymph nodes, each ~0.1cm, were palpated on the chest and there was normal chest auscultation. On abdominal palpation, there was no tenderness in any quadrant, but a marked pulsatile sensation was felt at the umbilicus. A loud bruit on expiration was heard following auscultation of the right lumbar and epigastric regions. His sexual maturity rating was at tanner 5, and the rest of his genitourinary (GU) exam was unremarkable. The patient was tested for secondary hypertension due to the abdominal bruit and Stage 2 hypertension. His lab results are shown below (Table 2).

**Table 2:** Laboratory values MCV: Mean corpuscular volume, BUN: Blood urea nitrogen, ALT: Alanine aminotransferase, AST: Aspartate aminotransferase, Aldo/PRA: plasma aldosterone to plasma renin activity, LC/MS/MS: Liquid chromatography with tandem mass spectrometry

	Laboratory Values	Reference Range
Hemoglobulin	16 g/dl	12–16 g/dl
Hematocrit	47%	36–49%
MCV	86.6 fl	78–98 fl
Platelet count	333 X 10^3^/uL	140–400 X 10^3^/uL
White blood count	6 X 10^3^/uL	4.5–13 X 10^3^/uL
Urinalysis	Normal	Normal
Sedimentation rate	2 mm/h	=< 15 mm/h
BUN	23 mg/dl	7–20 mg/dl
Creatinine	0.92 mg/dl	0.6–1.2 mg/dl
BUN/creatinine ratio	25 (prerenal picture)	9–25
Serum calcium	10.6 mg/dl	8.9–10.4 mg/dl
AST	24 U/L	56–234 U/L
ALT	26 U/L	8-46 U/L
Serum potassium	5.2 mmol/L	3.5–5.5 mmol/L
Plasma renin activity LC/MS/MS	4.22 ng/ml/h	0.25–5.82 ng/ml/h
Plasma renin activity Aldo/PRA ratio	2.8 ratio	0.9–28.9 ratio
Aldosterone, LC/MS/MS	12 ng/dl	=< 35 ng/dl
Total metanephrines and normetanephrines	174 pg/ml	=< 205 pg/ml
Drug screen	Marijuana detected	No drugs detected
Lipid panel	Normal	Normal

**Figure 1:**
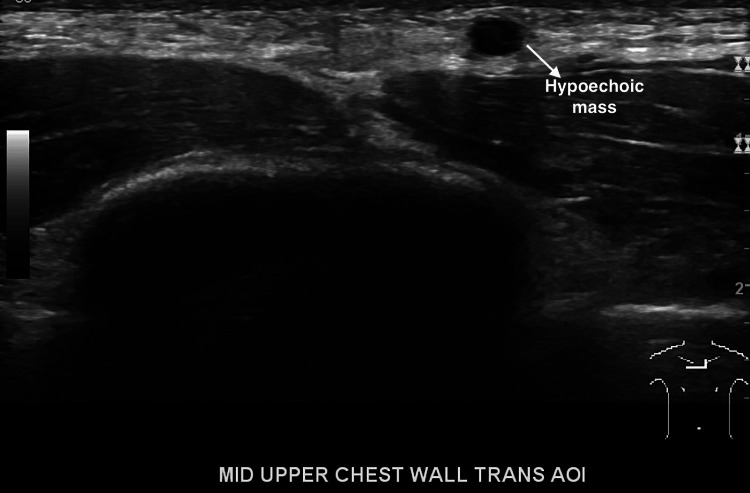
Chest ultrasound showing hypoechoic lesion suggestive of lymph nodes

**Figure 2:**
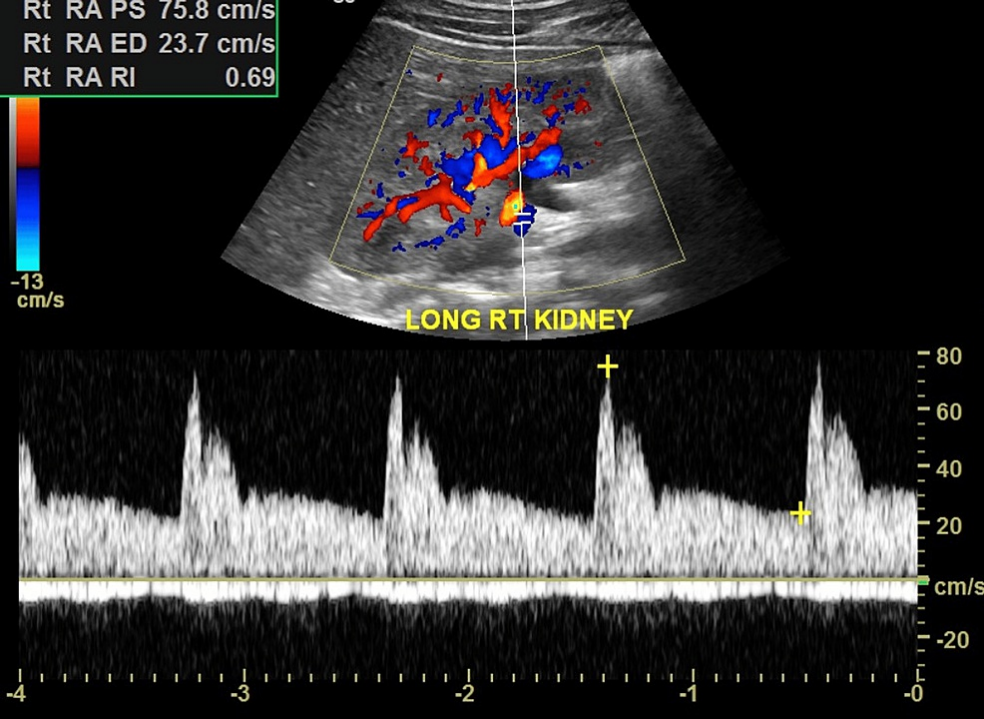
Renal duplex ultrasound of the right kidney The renal arteries are patent with normal blood flow. The peak systolic velocity is 75.8cm/s which is significantly less than 200cm/s, and hence Normal.

**Figure 3:**
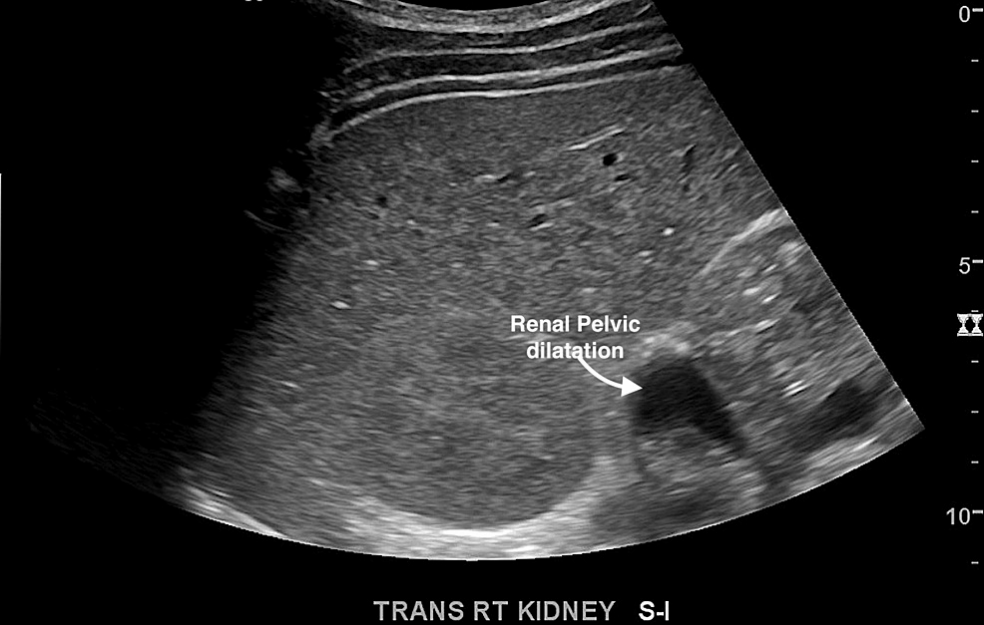
Duplex ultrasound (renal) of the right kidney The arrow shows the area of pelvic dilatation

**Figure 4:**
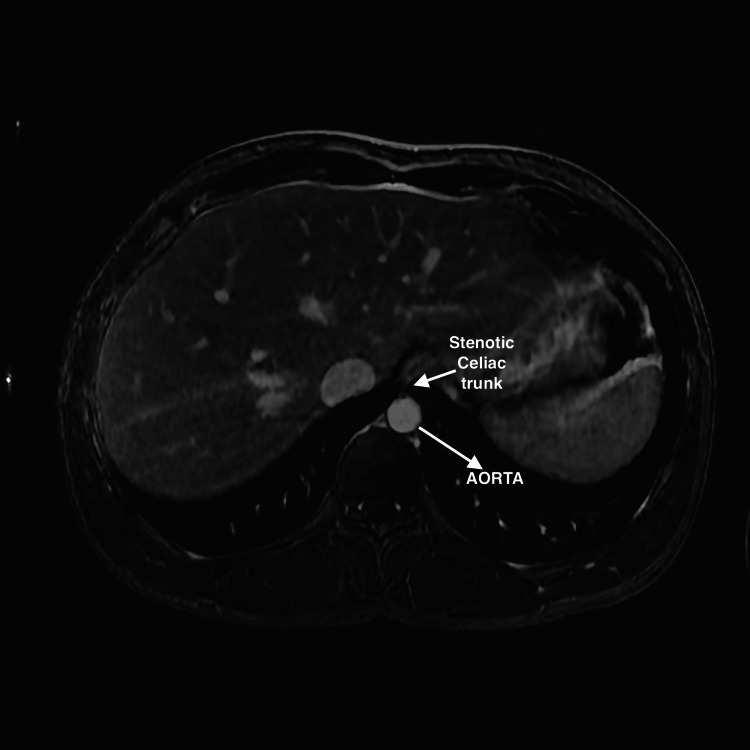
Coronal view of magnetic resonance angiography showing celiac artery compression by median arcuate ligament There is limited flow of contrast due to celiac artery compression causing stenosis. The celiac artery stems from the main aortic aorta

**Figure 5:**
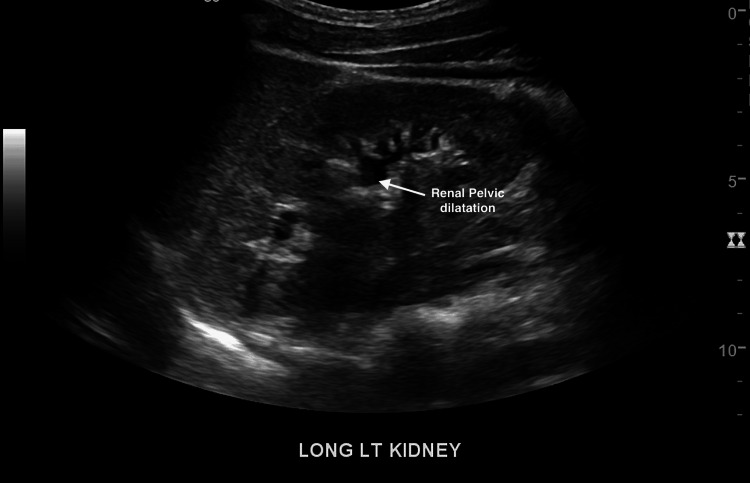
Abdominal ultrasound showing minimal renal pelvic dilatation of the left kidney

An electrocardiogram (EKG) showed normal sinus rhythm. The ultrasound (U/S) of the soft tissue of the chest wall confirmed anterior chest wall hypoechoic lesions and multiple small lymph nodes (Figure 1). A renal duplex ultrasound was negative for evidence of renal artery stenosis (no high velocities and patent renal arteries), and minimal renal pelvic dilatation (Figures 2, 3). Renal-aortic ratios (RAR) in the right and left kidneys were 0.5 and 0.6, respectively (normal RAR is less than 3.5). Magnetic resonance angiogram (MRA) with and without contrast confirmed celiac artery compression by the median arcuate ligament, causing moderate to severe stenosis of the proximal celiac arterial trunk with mild post-stenotic dilatation (Figure 4). There were no abdominal aortic aneurysms or significant atherosclerosis. The hepatobiliary system was normal with patent superior and inferior mesenteric arteries. Lastly, an abdominal ultrasound showed minimal bilateral pelvic dilatation (Figure 5).

**Figure 6:**
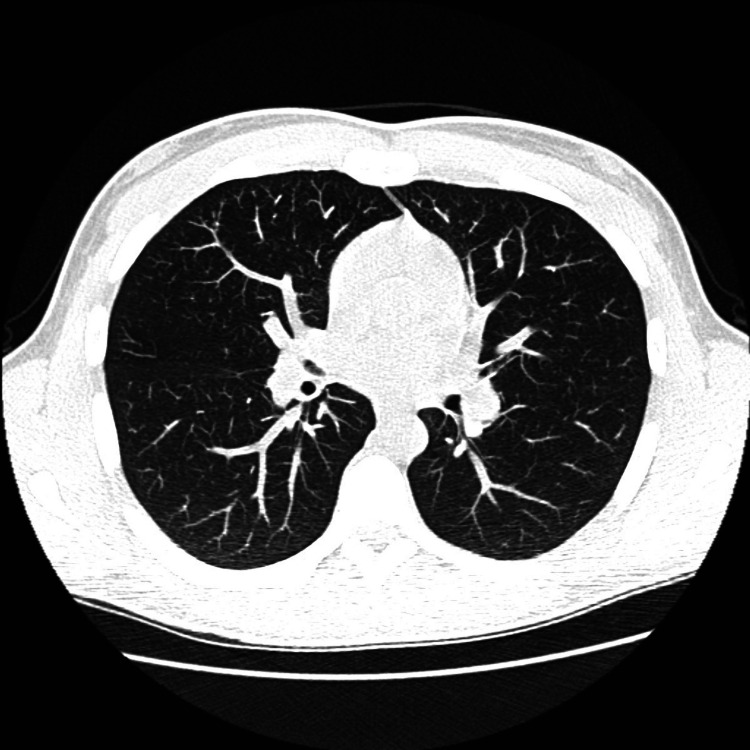
Normal chest CT scan

The patient was counseled about conservative measures to control his hypertension. He was referred to vascular surgery for possible minimally invasive decompression due to moderate-severe stenosis of the proximal celiac arterial trunk and significantly disproportionate weight loss from his CACS. The vascular surgery team did not pursue surgical intervention due to his low-risk profile. He was referred to the urology department due to bilateral pelvic dilatation. He was also counseled to discontinue marijuana use, as smoking is a strong complicating factor for his condition. His chest computed tomography (CT) scan was normal (Figure 6), and his subacute lymphadenopathy was attributed to the contiguous spread of his acne, as it subsequently resolved.

## Discussion

This case is diagnostically unique because of the patient's young age and vague symptoms. Due to the rarity of CACS and the lack of specificity of symptoms and signs, it is low on the list of differential diagnoses for chronic abdominal pain. Noteworthy, because CACS is a vascular pathology, there is ischemic pain; however, celiac ganglion compression can present as neuropathic pain [8]. This diagnosis is complex as 60% of patients with CACS do not have gastrointestinal angina due to insufficient collateral blood supply [9].

Although the superior mesenteric artery in this patient was patent on both U/S and MRA, moderate-severe stenosis of the celiac trunk, bloating, and weight loss should indicate the need for median arcuate release. Vascular imaging, including arteriography and duplex non-renal U/S, is required for a definitive diagnosis.

Catheter-based conventional arteriography will usually confirm obstruction of the lumen of the celiac axis and can evaluate flow dynamics or the presence of collateral blood supply [1]. However, it may fail to clearly outline the source of external compression (unlike CT, MRA, or duplex U/S) [1]. The common findings on a duplex U/S include visible external compression of the artery with expiration, increased flow velocities (>200 cm/second for the celiac artery, >275 cm/second for the superior mesenteric artery), and post-stenotic dilation [1]. The favorable outcomes for surgery include postprandial pain patterns, those aged 40-60 years, and weight loss >20 lbs., while unfavorable outcomes include atypical pain patterns with periods of remission, associated psychiatric disorders or alcohol abuse, and weight loss < 20 lbs [1]. Our patient has a mild prognosis due to the incidental nature of the presentation, young age, weight loss > 20 lbs., and an atypical pattern of sporadic bloating episodes that subsequently resolved. 

The available surgical interventions include open, laparoscopic, or robotic ligament release, celiac ganglionectomy*,* and celiac artery revascularization [10]. Surgical management should be done for symptomatic patients and asymptomatic patients with more than 50% stenosis of the celiac artery [11]. Median arcuate ligament release has an 85% success rate for post-operative symptom relief [12]. However, the recurrence rate is as high as 38% [13]. A minority of patients may continue to suffer debilitating symptoms despite treatment in the absence of other underlying diseases [11].

The differential diagnoses of postprandial abdominal pain can include cholecystitis, pancreatitis, gastroesophageal reflux disease, chronic intestinal ischemia, and gastric outlet obstruction [1]. The patient's unremarkable abdominal ultrasound, normal lipase levels, lack of chronic abdominal pain, or associated cyclical vomiting make these differentials unlikely.

## Conclusions

Celiac artery compression syndrome is rare in the pediatric population and can be an incidental finding. Pediatricians need to have this differential on their radars, especially when an abdominal bruit is found on clinical examination, as most patients may be otherwise asymptomatic. Due to its often vague but subtle presentation and low likelihood in younger individuals, there might be a delay in diagnosis and management if it is missed. Asymptomatic patients do not require surgery but need counseling to avoid risk factors like substance use, as well as “watchful waiting” on the recurrence of postprandial symptoms. Although symptomatic patients need surgery, with younger patients having a good prognosis, there is a risk of recurrence. Hence, it can be argued that delaying intervention until a pediatric patient is older is more suitable, especially in the absence of life-threatening symptoms. Therefore, this patient did not meet the criteria for surgery.
